# The MEP pathway in *Babesia orientalis* apicoplast, a potential target for anti-babesiosis drug development

**DOI:** 10.1186/s13071-018-3038-7

**Published:** 2018-08-06

**Authors:** Lan He, Pei He, Xiaoying Luo, Muxiao Li, Long Yu, Jiaying Guo, Xueyan Zhan, Guan Zhu, Junlong Zhao

**Affiliations:** 1 State Key Laboratory of Agricultural Microbiology, College of Veterinary Medicine, Huazhong Agricultural University, Wuhan, 430070 Hubei People’s Republic of China; 2 Key Laboratory for Development of Veterinary Diagnostic Products, Ministry of Agriculture, Huazhong Agricultural University, Wuhan, 430070 Hubei People’s Republic of China; 3 Key Laboratory of Preventive Veterinary Medicine in Hubei Province, The Cooperative Innovation Center for Sustainable Pig Production, Wuhan, 430070 Hubei China; 4 Department of Veterinary Pathobiology, College of Veterinary Medicine & Biomedical Sciences, Texas A&M University, College Station, Texas USA

**Keywords:** *Babesia orientalis*, Apicoplast, Isoprenoid, Methylerythritol phosphate (MEP) pathway, 1-deoxy-D-xylulose 5-phosphate reductoisomerase (DXR), Fosmidomycin

## Abstract

**Background:**

The apicomplexan parasite *Babesia orientalis*, the causative agent of water buffalo babesiosis in China, is widespread in central and south China, resulting in a huge economic loss annually. Currently, there is no effective vaccine or drug against this disease. *Babesia bovis* and *Plasmodium falciparum* were reported to possess an apicoplast which contains the methylerythritol phosphate (MEP) pathway inhibitable by fosmidomycin, suggesting that the pathway could serve as a drug target for screening new drugs. However, it remains unknown in *B. orientalis*.

**Methods:**

Primers were designed according to the seven MEP pathway genes of *Babesia microti* and *Babesia bovis*. The genes were cloned, sequenced and analyzed. The open reading frames (ORFs) of the first two enzyme genes, 1-deoxy-D-xylulose 5-phosphate synthase (BoDXS) and 1-Deoxy-D-xylulose 5-phosphate reductoisomerase (BoDXR), were cloned into the pET-32a expression vector and expressed as a Trx-tag fusion protein. Rabbit anti-rBoDXS and rabbit anti-rBoDXR antibodies were generated. Western blot was performed to identify the native proteins of BoDXS and BoDXR in *B. orientalis*. Fosmidomycin and geranylgeraniol were used for inhibition assay and rescue assay, respectively, in the *in vitro* cultivation of *B. orientalis*.

**Results:**

The seven enzyme genes of the *B. orientalis* MEP pathway (DXS, DXR, IspD, IspE, IspF, IspG and IspH) were cloned and sequenced, with a full length of 2094, 1554, 1344, 1521, 654, 1932 and 1056 bp, respectively. BoDXS and BoDXR were expressed as Trx-tag fusion proteins, with a size of 95 and 67 kDa, respectively. Western blot identified a 77 kDa band for the native BoDXS and a 49 kDa band for the native BoDXR. The drug assay results showed that fosmidomycin could inhibit the growth of *B. orientalis*, and geranylgeraniol could reverse the effect of fosmidomycin.

**Conclusions:**

*Babesia orientalis* has the isoprenoid biosynthesis pathway, which could be a potential drug target for controlling and curing babesiosis. Considering the high price and instability of fosmidomycin, further studies should focus on the screening of stable and cheap drugs.

## Background

Many members of the Apicomplexa, including *Plasmodium*, *Toxoplasma*, *Eimeria*, *Babesia* and *Theileria*, contain a unique, relic, non-photosynthetic plastid-like organelle termed apicoplast [1]. The apicoplast is a semi-autonomous organelle which normally contains a 35 kbp circular genome. There are three pathways in the apicoplast of *Plasmodium* and *Toxoplasma*, including the methylerythritol phosphate (MEP) pathway for isoprenoid biosynthesis, FAS pathway and heme operate. In *B. bovis* and *Babesia microti*, the isoprenoid biosynthesis MEP pathway is the only metabolic pathway in the apicoplast [2, 3]. Isopentenyl diphosphate (IPP) and dimethylallyl diphosphate (DMAPP) are two precursors to construct all the isoprenoids which are important for organisms. Isoprenoids comprise a large group of diverse intracellular metabolites with multiple cellular functions, including roles in membrane structure, cellular respiration and cell signaling [4].

In nature, there are two distinct biosynthetic routes to produce IPP and DMAPP: methylerythritol phosphate (MEP) pathway and a modified mevalonate (MVA) pathway. The mevalonic acid (MVA) pathway was thought to be the only IPP and DMAPP biosynthetic pathway for decades [5]. Mammals, including humans, use the MVA pathway that initiates from mevalonate, whilst eubacteria, cyanobacteria, plant chloroplasts and apicomplexan parasites exploit the methylerythritol phosphate (MEP) pathway that starts from pyruvate [6].

The MEP pathway is essential in eubacteria and protozoan but absent in humans and animals which could be the hosts of eubacteria and protozoan. The MEP pathway was discovered in the 1990s [7]. In this pathway, the reaction of all the steps are catalyzed by seven different enzymes, including 1-deoxy-D-xylulose 5-phosphate synthase (DXS), 1-deoxy-D-xylulose 5-phosphate reductoisomerase (DXR or IspC), 4-diphosphocytidyl-2*C*-methyl-D-erythritol cytidylyltransferase (IspD), 4-diphosphocytidyl-2*C*-methyl-D-erythritol kinase (IspE), 2*C*-methyl-D-erythritol-2, 4-cyclodiphosphate synthase (IspF), 1-hydroxy-2-methyl-2-(*E*)-butenyl-4-diphosphate synthase (IspG) and 1-hydroxy-2-methyl-2-(*E*)-butenyl-4-diphosphate reductase (IspH) [6]. The second enzyme DXR catalyzes step II of the conversion of 1-deoxy-D-xylulose 5-phosphate (DOXP) to 2-*C*-methyl-D-erythritol 4-phosphate (MEP), which is a target for fosmidomycin [8]. Fosmidomycin, a broad-spectrum antimicrobial agent showing the clinical promise of antimalarial drug, inhibits the isoprenoid biosynthesis by suppressing the activity of *P. falciparum* DXR, a rate-limiting enzyme of the MEP pathway, and the parasites will die for lack of isoprenoids (by blocking the conversion of DOXP to 2-*C*-methyl-D-erythritol 4-phosphate). Fosmidomycin is currently under Phase II clinical trials in combination with clindamycin and piperaquine against malaria [8–10].

*Babesia* spp. are tick-borne intraerythrocytic protozoans and cause babesiosis globally. *Babesia orientalis*, a recently identified species epidemic in China, causes water buffalo babesiosis. The parasite is transovarially transmitted by *Rhipicephalus haemaphysaloides* which is the only reported vector of *B. orientalis*, and water buffalo is the only reported host. As a member of the family Babesiidae, *B. orientalis* has a similar life-cycle with other babesiid species, including a sexual stage within *R. haemaphysaloides*, followed by an asexual stage in water buffalo RBC. Babesiosis has become one of the most important water buffalo diseases in central and south China [11]. However, there is no commercial vaccine or drug available to cure the disease. While the MEP pathway plays a critical role in the parasites and is reported to be a potential drug target, little information is available on the MEP pathway in *B. orientalis*. In this study, we proved that *B. orientalis* has the MEP pathway, and fosmidomycin can inhibit the growth of *B. orientalis*. The results demonstrate that the MEP pathway could serve as a potential target for screening anti-babesiosis drugs.

## Methods

### Parasites

*Babesia orientalis* (Wuhan strain) was previously isolated from Wuhan city, Hubei Province, China, and preserved in liquid nitrogen in the State Key Laboratory of Agricultural Microbiology, Huazhong Agricultural University, China.

Three one-year-old water buffaloes were purchased from a *Babesia*-free area. The buffaloes were tested by Giemsa staining and loop-mediated isothermal amplification (LAMP) to confirm that they were *Babesia* free/negative [12]. One water buffalo was used as a negative control. The other two water buffaloes were splenectomized 2 weeks before infection, and subcutaneously injected with 4 ml of *B. orientalis* positive blood (1% parasitemia). Blood was collected when the parasitemia of the infected buffaloes reached 3%.

### DNA extraction

Blood from the jugular vein of experimentally infected water buffaloes was collected in tubes containing EDTA (BD Vacutainer, USA). Genomic DNA was extracted from 200 μl of *B. orientalis*-infected blood using the QIAamp DNA Mini Kit (Qiagen, Shanghai, China) according to the manufacturer’s instructions. The DNA samples were used immediately or stored at -20 °C until use.

### RNA extraction and cDNA synthesis

Total RNA was extracted from the purified *B. orientalis* merozoites by using the TRIZOL reagent (Invitrogen, Shanghai, China) and treated with RNase-free DNaseI (TaKaRa, Dalian, China). RNA concentration was measured by Nanodrop 2000. The cDNA was prepared from 1 μg of the total RNA using PrimeScript^TM^ RT reagent Kit with gDNA eraser (TaKaRa, Dalian, China) according to the manufacturer’s instructions.

### Cloning of the seven enzyme genes of the MEP pathway

Primer pairs for the seven enzyme genes of the *B. orientalis* MEP pathway were designed based on the gene sequences of *B. microti* and *B. bovis* (Table 1). Complete sequences of the seven genes were amplified by conventional PCR from genomic DNA and cDNA separately. PCR products were purified and ligated into the cloning vector pDM19-T (TaKaRa, Dalian, China). Three positive colonies of each gene were sent for sequencing. The nucleotide sequences of the seven genes were submitted to GenBank.

**Table 1:** Primers used to amplify the seven MEP pathway enzyme genes of *Babesia orientalis*

Primer	Sequence (5′-3′)	Amplicon size (bp)
DXS-F	ATGGAGTTGTGTTGTAATC	2094
DXS-R	TTAGGATGCAAGGAATTGG	
DXR-F	ATGAATGCAGCAGTGAGTTTTTATG	gDNA 1554
DXR-R	TTAGTATGTGAAGCATTAATATATGTGATAG	cDNA 1371
IspD-F	ATGTCTGTCTGGTTGGTGCAATG	1344
IspD-R	TCACGGGAAGTATACCTCTTTTAG	
IspE-F	ATGAGGCATCTCCATGTGTG	1521
IspE-R	TCAAGAGATTAGGTCTGGAAGAAG	
IspF-F	ATGTTACTACGTTATCTTTCTATAACAG	654
IspF-F	TTATAAAGGATATTACTTGAGCTTC	
IspG-F	ATGACATCGTCAGACACCTTTG	1932
IspG-R	TCACATGCTTCCACATCTGG	
IspH-F	ATGGAGGAAAGACCGTTTACC	1056
IspH-R	TTATGTAGTTTTCCAGATTCCTATAATCG	

### Sequence analysis

The amino acid sequence of BoDXR was aligned with the amino acid sequences from other organisms, including *B. bovis*, *P. falciparum*, *Arabidopsis thaliana* and *Escherichia coli*. The alignment was performed by using the program MAFFT v7 [13] and then edited in BioEdit. Phylogenetic analysis of the aligned sequences was conducted using the neighbor-joining algorithm in MEGA 6 [14].

### Expression and identification of BoDXS and BoDXR

The open reading frame (ORF) of BoDXS was cloned into the pET-32a vector and expressed in *E. coli* BL21 (DE3). The recombinant protein was purified by affinity purification using a Ni-charged NTA column. Antisera against BoDXS were raised in rabbits.

In order to identify the native DXS in *B. orientalis*, the lysates of erythrocytes prepared from *B. orientalis* and *Babesia-*free water buffaloes (the negative control) (0.5 mg per lane) were separated by 12% SDS-PAGE, transferred onto nitrocellulose membrane (Millipore, Shanghai, China) and probed separately with rabbit serum raised against rBoDXS and pre-immune serum (1:100 dilution). The membranes were washed with PBST, and then incubated with the secondary antibody (1:5000, goat anti-rabbit IgG HRP, Beyotime, Shanghai, China). The expression and identification of BoDXR were conducted as previously described for BoDXS.

### *Babesia orientalis in vitro* cultivation and growth inhibition assay

Briefly, *B. orientalis* in the water buffalo erythrocytes was cultured in M199 media (Gibco Life Technologies, Shanghai, China) supplemented with 1% Albuman I (Gibco Life Technologies), 2% HB101 (Irvine Scientific, Shanghai, China), 1% L-glutamine (ATLANTA Biologicals, Shanghai, China), 2% Antibiotic/Antimycotic 100× (Corning, Shanghai, China) and 20% healthy water buffalo serum with 10% hematocrit (HCT) at 37 °C in a microaerophilous stationary phase (5% CO_2_, 2% O_2_, 93% N_2_). The cultures were sub-cultured every three days when the parasitemia reached 2–3%.

Drug stock solutions of fosmidomycin (Sigma-Aldrich, Shanghai, Chain) and diminazene aceturate (Sigma-Aldrich, Shanghai, Chain) were prepared in sterile water. Geranylgeraniol (Sigma-Aldrich, Shanghai, Chain) stocks were prepared in 100% ethanol. For the growth inhibition assay, *B. orientalis* cultures (total volume, 150 μl/well) were grown in 96-well flat-bottomed plates containing fosmidomycin in culture media. In the rescue experiments, the parasites were divided into five separate treatment groups (5 μM fosmidomycin, 5 μM fosmidomycin plus 5 μM geranylgeraniol, 5 μM geranylgeraniol plus 0.4 μM diminazene aceturate, 0.4 μM diminazene aceturate, and 0.1% ethanol). Diminazene aceturate was used as a positive control drug and ethanol was used as a blank control.

During the 72 h incubation of the plates, three smears were prepared from each well and the parasitemias were determined every 24 h by microscopy. Thin blood smears were air-dried, fixed with methanol, and stained with Giemsa (Sigma-Aldrich, Shanghai, China) according to the manufacturer’s instructions. The cultures were prepared in triplicate. The data were analyzed by GraphPad Prism 5 by one-way analysis of variance (ANOVA) followed by Turkey’s multiple comparison test. Error bars represent standard deviations.

## Results and discussion

### Characterization of the enzyme genes

**Fig. 1:**
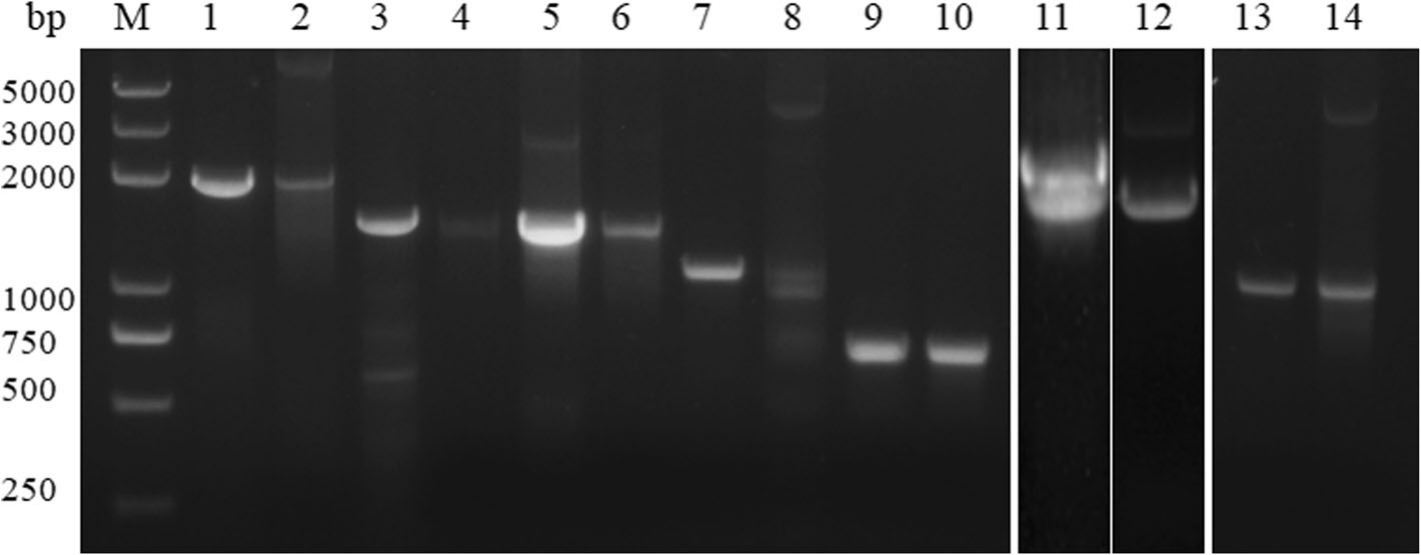
PCR amplification of the seven enzymes of the *B. orientalis* MEP pathway. Lane M; marker. Lanes 1, 3, 5, 7, 9, 11 and 13 were amplified from *B. orientalis* DNA. Lane 1: BoDXS; Lane 3: BoDXR; Lane 5: BoIspD; Lane 7: BoIspE; Lane 9: BoIspF; Lane 11: BoIspG; Lane 13: BoIspH. Lanes 2, 4, 6, 8, 10, 12 and 14 were amplified from *B. orientalis* cDNA, respectively. Lane 2: BoDXS; Lane 4: BoDXR; Lane 6 BoIspD; Lane 8: BoIspE; Lane 10: BoIspF; Lane 12: BoIspG; Lane 14: BoIspH

According to the reported genome sequences of *B. bovis* and *B. microti*, the genomes encode all the seven components of the isoprenoid pathway, suggesting that the parasites have the MEP pathway [3, 15]. In this study, the seven enzyme genes of the *B. orientalis* MEP pathway were amplified from both gDNA and cDNA and sequenced. The full lengths of DXS, DXR, IspD, IspE, IspF, IspG and IspH were 2094, 1554, 1344, 1521, 654, 1932 and 1056 bp, respectively (Fig. 1). These results indicated that all seven genes of the MEP pathway exist in the genome of *B. orientalis*. The nucleotide sequences of the DXS, DXR, IspD, IspE, IspF, IspG and IspH were submitted to the GenBank database under the accession numbers MH429606, MH429607, MH429608, MH448076, MH429609, MH429610 and MH429611, respectively.

**Fig. 2:**
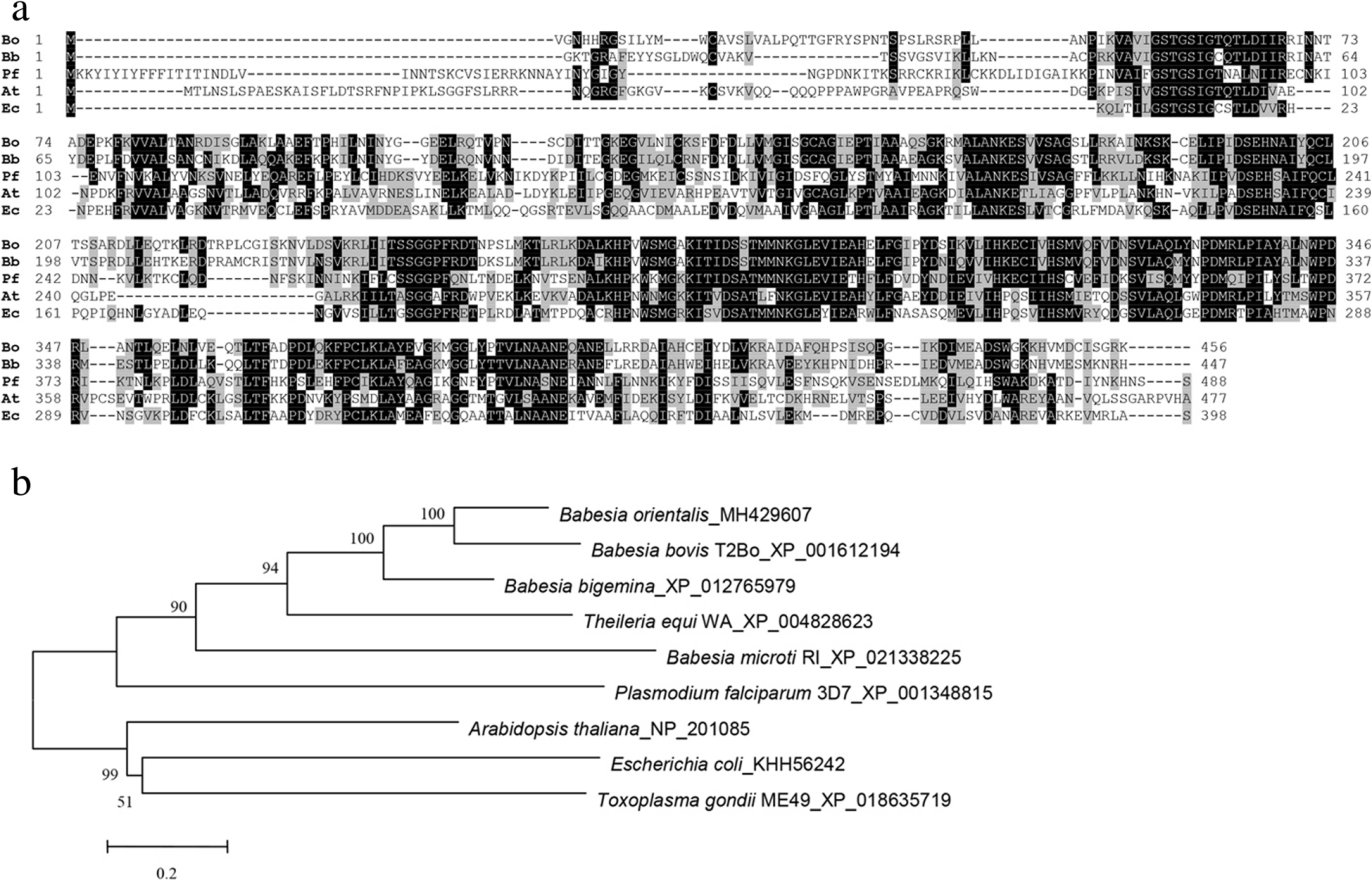
Multiple alignment and phylogenetic analysis of DXR amino acid sequence of DXR. **a** Multiple alignment of DXR aa sequences. Bo, *Babesia orientalis*; Bb, *Babesia bovis* (XP_001612194); Pf, *Plasmodium falciparum* (AAD03739); At, *Arabidopsis thaliana* (NP_201085); Ec, *Escherichia coli* (EGT67209). Similar and identical residues are shaded. Black shading indicates a similarity in four or more than four species; gray shading indicates a similarity in three species. **b** Neighbor-joining phylogenetic tree based on DXR aa sequences. Organism names and sequence accession numbers are indicated

BoDXS is intronless, encoding 667 aa with a predicted size of 77 kDa. BoDXR contains a 184 bp intron because the sequence obtained from cDNA is shorter than that of gDNA. It is the only intron gene in *B. orientalis* MEP pathway. BoDXR ORF is 1371 bp long, encoding 456 aa with a predicted size of 49 kDa. The amino acid sequence of BoDXR was aligned with the sequences of *B. bovis* (XP_001612194), *Plasmodium falciparum* (AAD03739), *Arabidopsis thaliana* (NP_201085) and *Escherichia coli* (EGT67209) (Fig. 2). The results indicated that DXR is highly conserved between these organisms and most close to *B. bovis*. For a better understanding of BoDXR and its homologs from related apicomplexans, a neighbor-joining tree was constructed. The results showed that BoDXR was most closely related to DXR from *B. bovis*. Sequence analysis indicated that BoDXR contains a NADB Rossmann superfamily domain and two DOXP redisom C superfamily domains. The NADB domain is found in numerous dehydrogenases of metabolic pathways, and it is responsible for specifically binding a substrate and catalyzing the enzyme reaction. The DOXP redisom C domain is the DXR C-terminus, which is also found in the DXR enzyme of bacteria and plants. It catalyzes the formation of 2-C-methyl-D-erythritol 4-phosphate (MEP) from 1-deoxy-D-xylulose-5-phosphate (DOXP) in the presence of NADPH.

### Characterization of native BoDXS and BoDXR

**Fig. 3:**
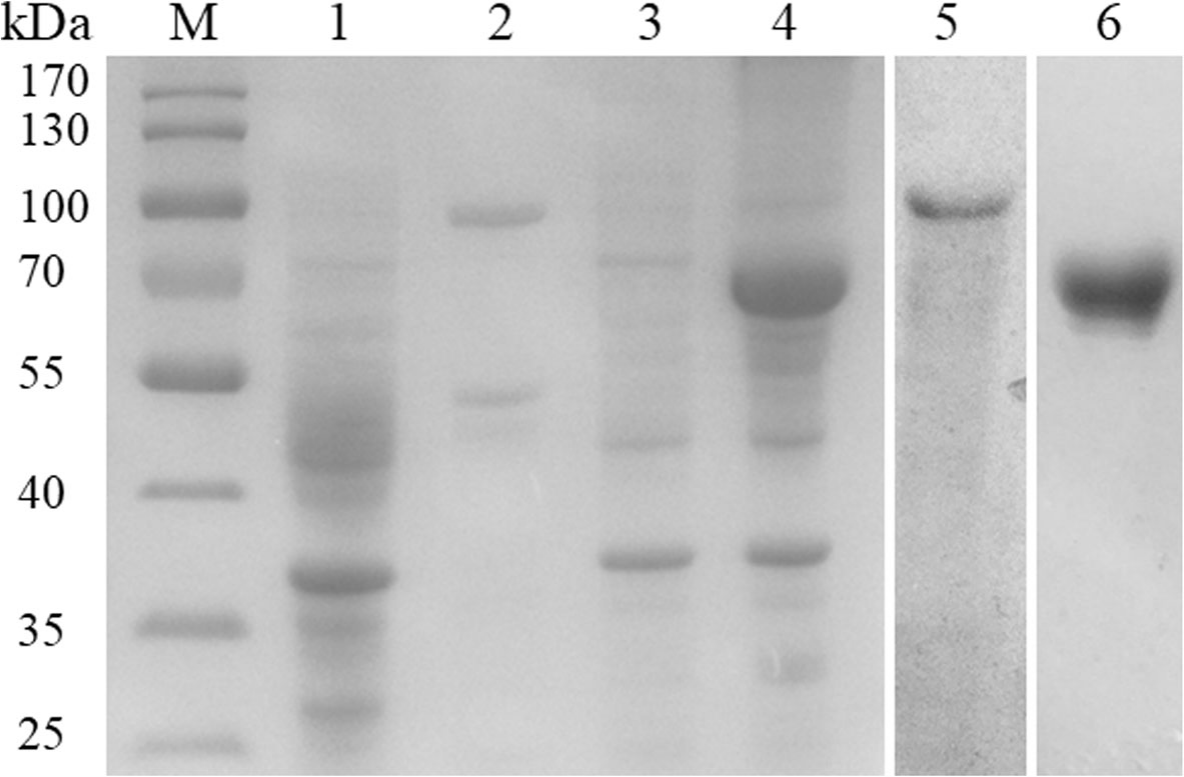
Expression and purification of rBoDXS and rBoDXR stained by coomassie brilliant blue. Lane M: molecular size marker; Lane 1: uninduced pET-32a-BoDXS; Lane 2: induced pET-32a-BoDXS; Lane 3: uninduced pET-32a-BoDXR; Lane 4: induced pET-32a-BoDXR; Lane 5: purified recombinant BoDXS fused with Trx-tag; Lane 6: purified recombinant BoDXR fused with Trx-tag

**Fig. 4:**
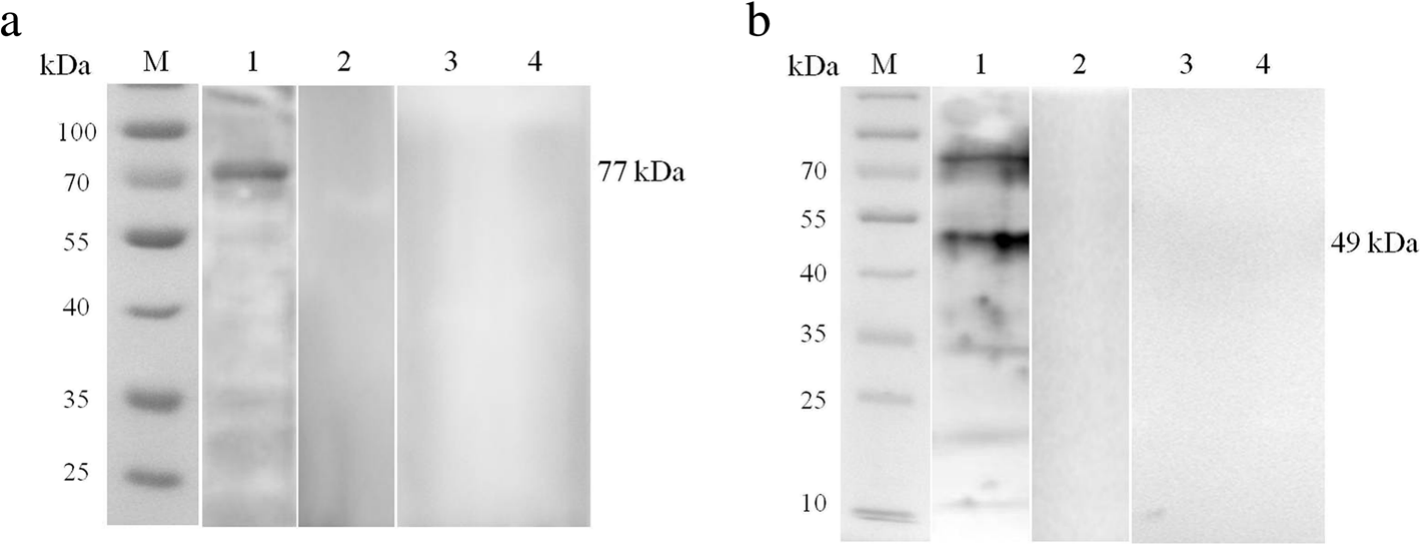
Identification of native BoDXS and BoDXR enzymes in *B. orientalis* merozoite lysates. **a** Identification of native BoDXS. Lysates of *B. orientalis*-infected water buffalo erythrocytes and lysates of uninfected water buffalo erythrocytes were both probed with the serum from rabbit immunized with rBoDXS (Lane 1 and Lane 3, respectively) and the pre-immune rabbit serum (Lane 2 and Lane 4, respectively). **b** Identification of native BoDXR. Lysates of *B. orientalis*-infected water buffalo erythrocytes and lysates of uninfected water buffalo erythrocytes were both probed with the serum from rabbit immunized with rBoDXR (Lane 1 and Lane 3, respectively) and the pre-immune rabbit serum (Lane 2 and Lane 4, respectively)

The initial enzyme DXS and the rate-limited enzyme DXR are the most important enzymes of the MEP pathway. 1-deoxy-D-xylulose 5-phosphate (DOXP), the product of DXS, also participates in thiamine (vitamin B1) biosynthesis and/or pyridoxine (B6) synthesis [16, 17], indicating that DXS is not a committed member of the MEP pathway. However, DXS is the first enzyme of the MEP pathway, and it catalyzes pyruvate and glyceraldehydes-3-phosphate to DOXP in the apicoplast of apicomplexan parasites. The second enzyme DXR, which is considered as the first committed enzyme of the MEP pathway, synthesizes 2-*C*-methyl-D-erythritol 4-phosphate (MEP) by intramolecular rearrangement and reduction of DOXP [17]. For biochemical characterization of BoDXS and BoDXR enzymes, the corresponding genes were over-expressed in *E. coli* and purified by Ni-NTA affinity chromatography. The purified rBoDXS and rBoDXR revealed the two bands of 95 and 67 kDa on 10% SDS-PAGE, including an additional 18 kDa Trx-tag (Fig. 3). Antibodies were generated in rabbit for detecting the native BoDXS and BoDXR. After the reaction of the rabbit anti-rBoDXS serum and rabbit anti-rBoDXR serum with *B. orientalis* lysates, the specific bands of 77 and 49 kDa were yielded, but not in the reaction with the lysates of the healthy bovine erythrocytes or pre-immune rabbit serum (Fig. 4). The results indicated that *B. orientalis* contains the native enzymes DXS and DXR.

### Fosmidomycin inhibits the growth of *B. orientalis*

The isoprenoid biosynthesis pathway generates isoprenoid precursors, and it is a promising chemotherapeutic target due to the difference between mammals and apicomplexan parasites in the seven enzymes of this pathway [8]. To date, the best characterized inhibitor of the MEP pathway in parasites is the phosphonic acid antibiotic fosmidomycin, a potential inhibitor of DXR by blocking the conversion of DOXP to 2-*C*-methyl-D-erythritol 4-phosphate [18]. Fosmidomycin is under Phase II clinical trials as a potential antimalarial chemotherapeutic agent in combination with clindamycin and piperaquine [8, 10]. The parasite *Babesia* is similar to *Plasmodium* in many aspects, and fosmidomycin was reported to be able to inhibit the intracellular growth of *B. divergens* at pharmacologically relevant concentrations [19]. However, there is no report about the inhibition of *B. orientalis* growth by fosmidomycin.

**Fig. 5:**
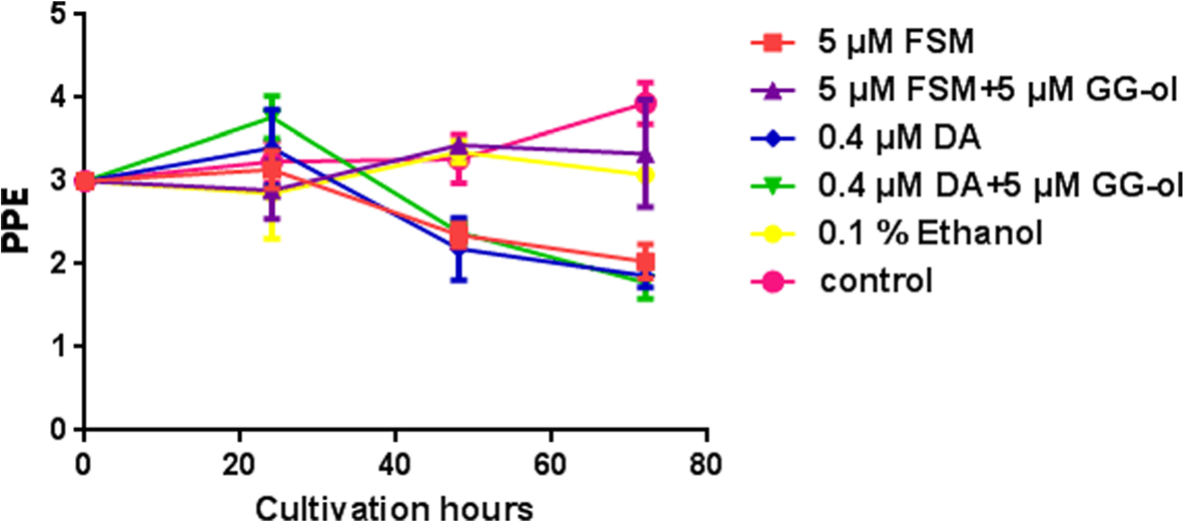
Fosmidomycin and geranylgeraniol treated parasites arrest during *in vitro* cultivation of *B. orientalis*. The parasites were treated separately with fosmidomycin (FSM), fosmidomycin plus geranylgeraniol (GG-ol), diminazene aceturate (DA), diminazene aceturate plus geranylgeraniol, and ethanol (control). Negative control without any treatment was also included. The parasitemias were determined by Giemsa staining every 24 h. PPE, percent parasitized erythrocytes

**Fig. 6:**
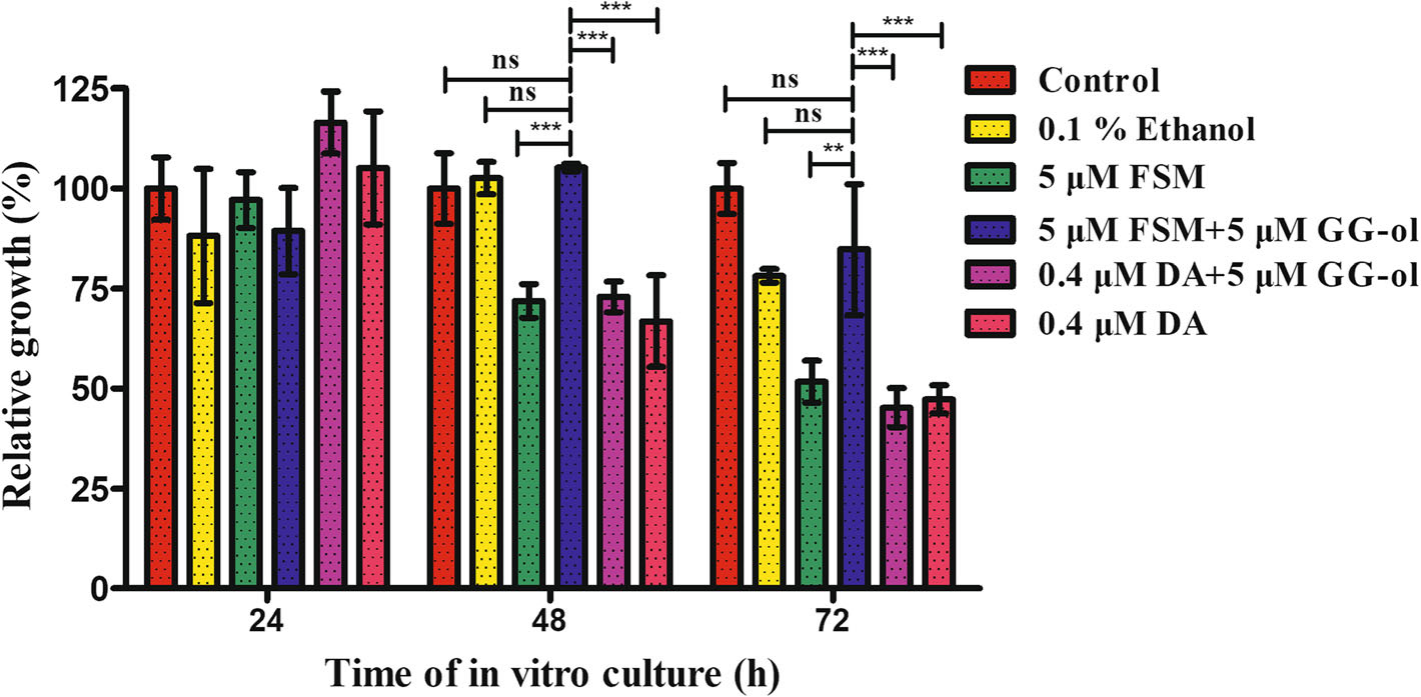
Efficacy of fosmidomycin on the growth of *B. orientalis*. *In vitro* cultured *B. orientalis* were treated separately with fosmidomycin (FSM), fosmidomycin plus geranylgeraniol (GG-ol), diminazene aceturate (DA), and diminazene aceturate plus geranylgeraniol. The growth of parasites was evaluated every 24 h for each treatment. 0.1% ethanol was used as the control. *P < 0.05, **P < 0.01, ***P < 0.001

In the present study, *B. orientalis* was cultured and treated separately with 5 μM fosmidomycin, 5 μM fosmidomycin plus 5 μM geranylgeraniol, 5 μM geranylgeraniol plus 0.4 μM diminazene aceturate, 0.4 μM diminazene aceturate, and 0.1% ethanol. Diminazene aceturate was used as a positive drug control. Ethanol was used as a control because geranylgeraniol was soluble in ethanol (treated concentration 0.1%). The parasitemias were examined by Giemsa staining and light microscopy every 24 h. The results indicated that fosmidomycin was effective in inhibition of *B. orientalis* growth after 24 h (Fig. 5), which is consistent with previous studies in *Babesia bovis* and *Plasmodium falciparum* [8, 15]. We found that both fosmidomycin and diminazene aceturate exhibited anti-babesiosis activity at 48 h and 72 h. The relative growth of fosmidomycin and negative controls were significant different at 48 h (ANOVA, *F*_(3, 8)_ = 23.07, *P* = 0.0015) and 72 h treatment (ANOVA, *F*_(3, 8)_ = 73.21, *P* < 0.0001). However, there was no notable difference between fosmidomycin plus geranylgeraniol and control groups (both negative control and 0.1% ethanol) (Fig. 6). The effects of fosmidomycin were reversed by medium supplemented with a downstream isoprenol geranylgeraniol, confirming that they are specific to isoprenoid blockade.

*Plasmodium falciparum* was reported to utilize the non-mevalonate isoprenoid biosynthesis in the apicoplast which is essential to the parasites [8, 20, 21]. Our previous work demonstrated that *B. orientalis* contains an apicoplast with a 34 kpb circular genome [22], but whether the isoprenoid biosynthesis pathway exists in this parasite remains unknown. In this study, the inhibitory effect of fosmidomycin and the rescue effect of geranylgeraniol on *B. orientalis* indicated the presence of the substrate of this pathway, further suggesting that the MEP pathway is functional and required for the intraerythrocytic growth of *B. orientalis* merozoites, which is similar to that of *B. bovis* and *P. falciparum* [15, 21]. The effectiveness of fosmidomycin may be attributed to the presence of a new permeability pathway of erythrocytes, which allows the transport of fosmidomycin across the cell membrane to effectively inhibit the growth of apicomplexan parasites including *Plasmodium* and *Babesia* which reside within erythrocytes [19, 23]. The MEP pathway was also reported in *Theileria parva*, but fosmidomycin showed no inhibitory effect even at a high concentration of 400 μM [24].

## Conclusions

In the present study, we cloned and sequenced the seven enzyme genes of the MEP pathway in *B. orientalis*. The native proteins of BoDXS and BoDXR were also identified, which demonstrated that the parasite contains the initial enzyme and the rate-limited enzyme of the MEP pathway. Additionally, the growth of *B. orientalis* could be inhibited by fosmidomycin while the effects of fosmidomycin could be reversed by medium supplemented with geranylgeraniol. These results indicated the existence of the isoprenoid biosynthesis pathway in *B. orientalis*, which could be a potential drug target for screening anti-babesiosis drugs. Further research should focus on the biological function of the MEP pathway in *B. orientalis* and the screening of new drugs using the *E. coli* model due to its identical isoprenoid biosynthesis pathway with apicomplexan parasites and easiness in manipulation.

## Abbreviations

DMAPP: Dimethylallyl diphosphate
DOXP: 1-deoxy-D-xylulose 5-phosphate
DXR or IspC: 1-deoxy-D-xylulose 5-phosphate reductoisomerase
DXS: 1-deoxy-D-xylulose 5-phosphate synthase
IPP: Isopentenyl diphosphate
IspD: 4-diphosphocytidyl-2*C*-methyl-D-erythritol cytidylyltransferase
IspE: 4-diphosphocytidyl-2*C*-methyl-D-erythritol kinase
IspF: 2*C*-methyl-D-erythritol-2, 4-cyclodiphosphate synthase
IspG: 1-hydroxy-2-methyl-2-(*E*)-butenyl-4-diphosphate synthase
IspH: 1-hydroxy-2-methyl-2-(*E*)-butenyl-4-diphosphate reductase
MEP pathway: Methylerythritol phosphate pathway
MEP: 2-*C*-methyl-D-erythritol 4-phosphate (methylerythritol phosphate)
MVA: Pathway: mevalonate pathway
